# Disparities in Cervical Cancer Screening with HPV Test among Females with Diabetes in the Deep South

**DOI:** 10.3390/cancers13246319

**Published:** 2021-12-16

**Authors:** Cassidi C. McDaniel, Hayleigh H. Hallam, Tiffany Cadwallader, Hee-Yun Lee, Chiahung Chou

**Affiliations:** 1 Department of Health Outcomes Research and Policy, Harrison School of Pharmacy, Auburn University, Auburn, AL 36849, USA; cnc0027@auburn.edu (C.C.M.); hhh0010@auburn.edu (H.H.H.); tjc0049@auburn.edu (T.C.); 2 School of Social Work, The University of Alabama, Tuscaloosa, AL 35487, USA; hlee94@ua.edu; 3 Department of Medical Research, China Medical University Hospital, Taichung 40447, Taiwan

**Keywords:** cervical cancer, cancer screening, HPV, diabetes, health disparities

## Abstract

**Simple Summary:**

Diabetes is linked with poorer cervical cancer prognosis, and people residing in the Southern region of the U.S. are disproportionately diagnosed with diabetes and cancer. The HPV test was recently recognized as the preferred method of cervical cancer screening by the American Cancer Society. Through our observational study, we sought to investigate the HPV testing behaviors among females with and without diabetes across the U.S. Our nationally representative estimates reveal that less than half of females reported HPV testing, and females with diabetes in the Deep South have the lowest rates of HPV testing. Various risk factors were identified to significantly lower the odds of HPV testing, including a diabetes diagnosis, older age, living in the Southern region of the U.S., and absence of certain comorbidities. The lower rates of HPV testing among females with diabetes, especially those living in the Deep South, leave these populations vulnerable to cervical cancer.

**Abstract:**

Background: Due to diabetes being linked with poorer cervical cancer prognosis, this study aimed to evaluate HPV testing behaviors among females with and without diabetes across the U.S. by geographic area in 2016, 2018, and 2020. Methods: This cross-sectional study used the Behavioral Risk Factor Surveillance System (BRFSS) from 2016, 2018, and 2020. The study population included females aged 25–69 years old, stratified by self-reported diabetes status. The primary outcome measure was cervical cancer screening behavior, which was evaluated by self-reported HPV test uptake/receipt (yes/no). Results: A total of 361,546 females from across the U.S. were sampled. Within the study population combined from all study years, the overall likelihood of receiving an HPV test was significantly lower among females with diabetes [37.95% (95% CI: 36.87–39.04)] compared to those without diabetes [46.21% (95% CI: 45.84–46.58)] (*p* < 0.001). Screening rates with HPV tests were lowest among females with diabetes in the South in 2016 (29.32% (95% CI: 26.82–31.83)), 2018 (39.63% (95% CI: 36.30–42.96)), and 2020 (41.02% (95% CI: 37.60–44.45)). Conclusions: Females with diabetes are screening with HPV tests less frequently than females without diabetes, and females living in the South, particularly states in the Deep South, report the lowest rates of HPV testing.

## 1. Introduction

Diabetes continues to be a significant health challenge in the U.S., where the prevalence has consistently increased over time [1]. Some project that by 2060, the number of diagnosed diabetes cases in adults will almost triple, with the respective percent prevalence doubling [2]. With the future bringing drastically increased diabetes cases, an additional concern arises: cancer. Diabetes and cancer are disproportionately experienced by people residing in the Southern region of the U.S., now referred to as the “diabetes belt” [3] and the “cancer belt” [4]. Here, these states will be referred to as the “Deep South,” including Alabama, Georgia, Louisiana, Mississippi, South Carolina, and Tennessee [5,6]. In the Deep South, the high rates of diabetes and cancer warrant investigation of potential health disparities.

The proposed relationship between diabetes and cancer has drawn experts’ interest for a long time, as the two are frequently co-morbid conditions [7,8]. Although the specific relationship is rather complex, similar risk factors for the two serve as the most probable indication [7,9]. The connections present concerns for increased cancer incidence and mortality [10]. Recent studies have shown a higher incidence of female cancers in persons with diabetes, where diabetes and cervical cancer have been strongly correlated [11]. Diabetes is an important factor when evaluating cervical cancer prognoses and has been shown to lead to lower survival rates in those who develop cancer [12]. Additionally, high blood glucose levels may increase the risk of developing certain cervical lesions associated with human papillomavirus (HPV) [13,14]. Therefore, prevention of cervical cancer development and promotion of early detection practices are essential in the U.S., particularly among females with diabetes.

Cervical cancer is burdensome to females across the U.S. A total of 12,733 females were newly diagnosed with cervical cancer in 2018 alone, and the cervical cancer mortality was 4138 [15]. In 2021, the American Cancer Society (ACS) expects these estimates to increase to approximately 14,480 new diagnoses and 4290 deaths [16]. The cervical cancer incidence also differs based on geographic area in the U.S., and the Deep South is an area with notably high rates of cervical cancer [17]. The cervical cancer burden also affects patients directly through significant increases in their healthcare spending, where national estimates revealed that the annual healthcare spending among a female with cervical cancer was double that of a female without cervical cancer [18]. Additionally, cervical cancer has been shown to negatively impact patients’ humanistic outcomes, such as decreased quality of life, increased activity limitations, and increased depression severity [18].

While the burden of cervical cancer remains alarming, the number of cervical cancer cases has declined since the introduction of the HPV vaccine in 2006 [19,20,21] and the FDA approval of the new 9-valent HPV vaccine in 2014 [22]. The Centers for Disease Control and Prevention (CDC) recommends that teens and young adults aged 11–26 receive either two or three doses to complete the HPV vaccine series [23]. However, initiation and completion of the HPV vaccine series remain low [24]. In the U.S. as of 2016, it was reported that only 65% of female adolescents aged 13–17 years had initiated the HPV vaccine series with just one dose, and only 50% had completed the recommended series [25]. As of 2018, slightly more than half of female adolescents were not fully vaccinated with the HPV vaccine series [26]. This indicates that many female adolescents may be entering adulthood without being fully vaccinated against HPV, leaving them more at risk of contracting the virus and developing HPV-related cervical cancer. Further, uptake of the HPV vaccine is especially problematic in the Deep South, where the rates of HPV vaccine completion in Alabama, Georgia, Louisiana, Mississippi, South Carolina, and Tennessee fall lower than the national completion rates [27]. In particular, South Carolina and Mississippi had extremely low vaccination rates in 2018 at 33% and 35.1%, respectively [27]. Challenges with HPV vaccine uptake in the Deep South may be affected by limited HPV knowledge, beliefs about vaccines, or education from healthcare professionals [28]. Because of the persistent low uptake and completion of the HPV vaccine series, cervical cancer continues to be a concern [29], and many females are left in need of alternative prevention methods.

Cancer screening serves as an effective tool for early cancer detection, and uptake of cervical cancer screening practices can have public health implications on cancer incidence and mortality outcomes [21]. The two general methods used for cervical cancer screening are the Pap and HPV tests, which are taken individually or together via co-testing through the same method of collecting cervical cells [30]. The Pap test has made significant progress in detecting cervical cancer since its introduction in the 1950s [31]. However, the ACS has recently recognized the primary HPV test as the preferred method of screening for cervical cancer, which is recommended among females aged 25–65 [30].

HPV is now recognized as the cause for most cervical cancer diagnoses [16], so testing for HPV can help determine potential outcomes related to cervical cancers and open doors to educate females about decreasing their risk of contracting HPV. The HPV test is FDA approved and is more sensitive to HPV strains 16 and 18 that are likely to cause HPV-associated cancers [30]. The HPV test can be used alone at a five-year interval, decreasing the need for co-testing with Pap. Utilizing HPV testing as the primary screening method for cervical cancers could mean a longer interval between testing and more specific protocols for positive tests compared to the Pap test [32].

Participation in proper cervical cancer screenings among persons with diabetes could allow for the best chance at early detection and a more favorable prognosis. Despite this, there have been limited studies published that address these behaviors in this population. Through systematic review and meta-analysis, one study found that females with diabetes were significantly less likely to screen for cervical cancer than females without diabetes [33]. However, researchers acknowledged that future studies should evaluate diabetes status contribution to other factors at the patient, provider, and system levels. Additionally, with the recently updated ACS guidelines introducing a preference for primary HPV testing, gaps in research for HPV testing and its use among females with diabetes must be investigated.

Our study aims to evaluate cervical cancer screening behaviors among females with and without diabetes across the U.S. by investigating their HPV testing practices in 2016, 2018, and 2020. Due to the disproportionately high diabetes rates and low utilization of cervical cancer screening practices in various regions of the U.S. [3,34], estimates of HPV testing are evaluated by geographic area, with particular focus on states located in the Deep South. Our findings will serve as critical contributions to populations with diabetes so that appropriate initiatives to increase screening rates may be explored.

## 2. Materials and Methods

### 2.1. Study Design and Data Source

This cross-sectional study used the Behavioral Risk Factor Surveillance System (BRFSS) data to evaluate cervical cancer screening behaviors among females in the U.S. The BRFSS is a cross-sectional telephone survey administered by the CDC that provides standardized questions regarding risk behaviors and preventive healthcare practices among a nationally representative sample [35]. Additionally, data weighting is used for the survey design and iterative proportional fitting to remove bias from the sample. The BRFSS survey includes an annual standard core, a biannual rotating core, optional modules, and state-added questions [35]. For this study, the survey questions of interest pertaining to cervical cancer screening behaviors were only present in core modules for even-numbered years. With 2016 being the first year of BRFSS data where HPV testing questions were available from all states, our data is from 2016, 2018, and 2020 samples, which comprehensively includes the national BRFSS data available to date that capture cervical cancer screening behaviors in core modules.

### 2.2. Study Population

The inclusion criteria remained broad to maximize external validity, so the study population included adult females aged 25–69. This study population was chosen by following the ACS guidelines for primary HPV testing to include survey respondents that were the correct biological sex for the respective cancer screening and within the age range of 25–65 years old [30], which required the inclusion of BRFSS five-year age brackets from 25–29 to 65–69. Females with prior hysterectomy were excluded. This study population was stratified by self-reported diabetes status, which was measured with the question, “Has a doctor, nurse, or other health professional ever told you that you have diabetes?” We categorized those who responded “yes” or “yes, gestational diabetes” as females with diabetes, those who responded “no,” “no, prediabetes or borderline diabetes,” or “don’t know/not sure” as females without diabetes, and those who “refused” or did not answer the question as missing data. We chose to include females considered to have gestational diabetes because about 50% of this population in the U.S. typically develop type 2 diabetes, which is essential to consider given our population of focus [36].

### 2.3. Outcome Measures

The primary outcome measure was cervical cancer screening behavior, which was evaluated by self-reported HPV test uptake/receipt. The question used was, “An HPV test is sometimes given with the Pap test for cervical cancer screening. Have you ever had an HPV test?” The outcome was categorized as yes for those who responded “yes” to HPV test screening, no for those who responded “no” or “don’t know/not sure,” and missing for those who “refused” or did not answer the question.

### 2.4. Statistical Analysis

Statistical analyses were performed using SAS Version 9.4 (SAS Institute, Cary, NC, USA). Analyses utilized the appropriate survey procedures in SAS to obtain accurate estimates representing the U.S. population, such as ‘proc surveyfreq’ and ‘proc surveylogistic’ [37]. Weight, cluster, and strata variables were included in analyses of questions from core modules recommended by BRFSS to account for complex survey design [37]. The sample size was reported as unweighted (*n*) to represent the actual number of BRFSS respondents and weighted (weighted *n*) to represent the population after considering the BRFSS sampling design [35,38].

The study population was stratified by diabetes diagnosis to estimate differences in cervical cancer screening behaviors. Data from 2016, 2018, and 2020 were reported separately to show trends in cancer screening behaviors over time. These behaviors were further estimated for geographical area differences in the South, Midwest, West, and Northeast. These regions were determined using the U.S. Census Regions and Divisions with State FIPS code [39]. U.S. territories (Guam, Puerto Rico, and the Virgin Islands) were included in the study population but were not included in the calculation of these regional estimates. Females with diabetes living in the Deep South (i.e., Alabama, Georgia, Louisiana, Mississippi, South Carolina, and Tennessee) were then measured for their cervical cancer screening practices. Residential status was determined using the responses to the State FIPS Code used for record identification.

To detect differences in characteristics and HPV testing practices between females with and without diabetes, we performed Rao–Scott chi-square tests and estimated respective percentages and 95% confidence intervals (95% CI). For characteristics presented in Table 1, data from all years were joined to reflect one population and its relevant covariates. HPV testing practices were analyzed as national estimates, regional estimates by geographic location, and state estimates in the Deep South. A logistic regression model was used to predict the odds of HPV testing while adjusting for covariates. Comorbid health conditions were included as covariates based on the prior literature demonstrating lower rates of cervical cancer screening (via Pap and Pap-HPV co-testing) among females with comorbid conditions [40]. The reference group selected for each variable was the category with the highest frequency, except for metropolitan status (category representing rural selected as reference group) and age (youngest age selected as reference group) variables. Adjusted odds ratios (aOR) and 95% CI are reported. Significance was set at alpha <0.05, and hypothesis tests were two-sided. The study protocol was approved by the Institutional Review Board at the primary authors’ institution.

## 3. Results

### 3.1. Study Population Characteristics

In 2016, 2018, and 2020 BRFSS samples, 361,546 females aged 25–69 years old met inclusion criteria for the study population. A total of 41,442 females self-reported diabetes, and the remaining 320,104 self-reported not having diabetes (Table 1). The distribution of race/ethnicity was significantly different between females with and without diabetes (*p* < 0.001). The South had the highest number of respondents, representing 38.90% (95% CI: 37.81–39.98) of females with diabetes and 35.74% (95% CI: 35.48–36.00) of females without diabetes. More females with diabetes reported their health status as either “fair” or “poor” [27.91% (95% CI: 26.91–28.91) and 12.03% (95% CI: 11.19–12.86), respectively] than females without diabetes [10.18% (95% CI: 9.94–10.41) and 2.60% (95% CI: 2.49–2.72), respectively] (*p* < 0.001).

### 3.2. HPV Testing: National and Regional Estimates

**Figure 1:**
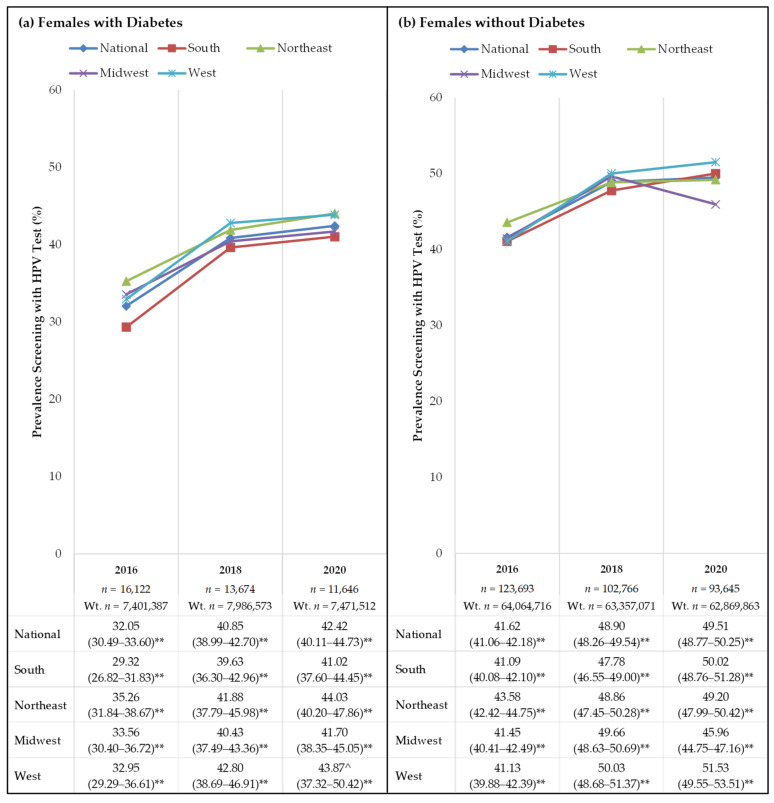
(**a**) HPV Testing Behaviors among Females with Diabetes across Geographic Areas of the U.S.; (**b**) HPV Testing Behaviors among Females without Diabetes across Geographic Areas of the U.S. Weighted percentages (Weighted % (95% CI)) are presented for females who self-reported screening for cervical cancer with HPV test; age parameters follow the American Cancer Society’s guidelines for screening. Wt. *n* = weighted sample size. ^^^ Use caution when interpreting this sub-group prevalence estimate; 95% confidence interval width > 10. ** *p* < 0.05; chi-square tests detected significant differences in prevalence screening with HPV test between females with and without diabetes.

Within the study population combined from all study years, the overall likelihood of receiving an HPV test was significantly lower among females with diabetes [37.95% (95% CI: 36.87–39.04)] compared to those without diabetes [46.21% (95% CI: 45.84–46.58)] (*p* < 0.001). Looking at each year separately, Figure 1 displays the prevalence of cervical cancer screening through HPV testing rates in females with and without diabetes nationally and within U.S. regions (South, Northeast, Midwest, and West). Nationally, females with diabetes screened significantly less compared to those without diabetes in all years [2016: 32.05% (95% CI: 30.49–33.60) vs. 41.62% (95% CI: 41.06–42.18); 2018: 40.85% (95% CI: 38.99–42.70) vs. 48.90% (95% CI: 48.26–49.54); 2020: 42.42% (95% CI: 40.11–44.73) vs. 49.51% (95% CI: 48.77–50.25), respectively] (*p* < 0.001 for all). Across the entire study population, screening rates with HPV tests were lowest among females with diabetes in the South in 2016 (29.32% (95% CI: 26.82–31.83)), 2018 (39.63% (95% CI: 36.30–42.96)), and 2020 (41.02% (95% CI: 37.60–44.45)).

### 3.3. HPV Testing in the Deep South

**Figure 2:**
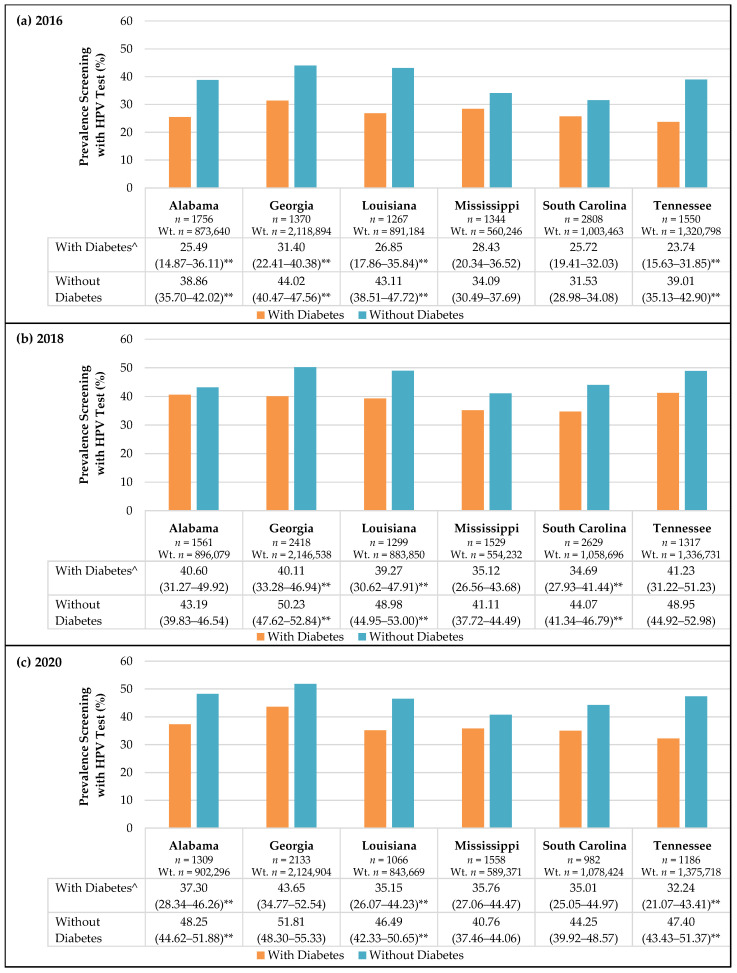
HPV Testing Behaviors of Females with and without Diabetes among States in the Deep South in (**a**–**c**) 2020. Weighted percentages (Weighted % (95% CI)) are presented for females who self-reported screening for cervical cancer with HPV test; age parameters follow the American Cancer Society’s guidelines for screening. Wt. *n* = weighted sample size. ^^^ Use caution when interpreting prevalence estimates within this sub-group; unweighted frequency <50 or 95% confidence interval width >10. ** *p* < 0.05; chi-square tests detected significant differences in prevalence screening with HPV test between females with and without diabetes in this state.

Figure 2 shows the prevalence of HPV screening among females with and without diabetes in the Deep South region of the U.S. Females with diabetes consistently screened with HPV test less often than females without diabetes. In 2016, females with diabetes had significantly lower rates of HPV screening compared to those without diabetes in Alabama, Georgia, Louisiana, and Tennessee (*p* < 0.05 for all). In 2018, females with diabetes had significantly lower rates of HPV screening compared to those without diabetes in Georgia, Louisiana, and South Carolina (*p* < 0.05 for all). In 2020, females with diabetes had significantly lower rates of HPV screening compared to those without diabetes in Alabama, Louisiana, and Tennessee (*p* < 0.05 for all).

### 3.4. Factors Associated with HPV Testing Behaviors

Table 2 shows the percentage of women screening with the HPV test across various factors along with the odds of HPV testing while adjusting for covariates. Diabetes was significantly associated with lower odds of HPV testing (aOR: 0.934, 95% CI: 0.886–0.985). Compared to females identifying as White, those identifying as Black only/non-Hispanic, American Indian/Alaska Native, multiracial/non-Hispanic, and Hispanic had greater odds of screening (aOR: 1.352, 95% CI: 1.287–1.420; aOR: 1.150, 95% CI: 1.007–1.314; aOR: 1.408, 95% CI: 1.268–1.563; aOR: 1.221, 95% CI: 1.155–1.291; respectively), whereas Asian/non-Hispanic females had lower odds (aOR: 0.570, 95% CI: 0.514–0.631). Living in the Northeast or West was significantly associated with higher odds of HPV testing compared to living in the South (aOR: 1.138, 95% CI: 1.093–1.184 and aOR: 1.115, 95% CI: 1.066–1.167; respectively). Compared to females aged 25–29, females aged 30–34 had higher odds of HPV testing, but females 40 years and older had lower odds of HPV testing, where the odds of testing decreased with increasing age. Compared to females not living in metropolitan statistical areas (MSA), females living in and around MSA exhibited significantly higher odds of screening. Socioeconomics, including lower educational status, lower income categories, and lack of insurance, were associated with lower odds of HPV testing. Lastly, the odds of HPV testing were significantly higher in more recent years.

## 4. Discussion

To better understand current cervical cancer screening behaviors across the U.S., this study investigated HPV testing practices among females with and without diabetes. Overall, HPV testing rates did not meet the target cervical cancer screening rate of 84.3% proposed by Healthy People 2030 [41]. The Healthy People 2030 target cervical cancer screening rate was proposed within the following objective to promote preventive care for cancer: “Increase the proportion of females who get screened for cervical cancer” [41]. However, our study findings demonstrate that females with diabetes overwhelmingly screen less with the HPV test, which points to a potential health disparity. In 2016, 2018, and 2020, nearly 68%, 59%, and 58% of females with diabetes and within the screening age were not screening for cervical cancer with an HPV test, respectively. Even though utilization of HPV testing increased across time, a large population of females with diabetes remains at risk of undetected cervical cancer and in need of the recommended HPV testing.

The strength and significance of this study are found in the timely investigation of cervical cancer screening with the HPV test based on recent guideline updates from the ACS in 2020, along with the nationally representative nature of the findings. The novelty of this study lies in our investigation of HPV testing behaviors between females with and without diabetes. We found diabetes to be significantly associated with a lower likelihood of HPV testing, which is similar to previous studies that found strong associations among females with diabetes and lower cervical cancer screening rates [42,43]. An important distinction is that these previous studies were conducted prior to the ACS guideline update in 2020, so these studies conceptualized cervical cancer screening through Pap testing alone [42,43]. Our study builds on the existing evidence-base by demonstrating that the negative association between diabetes and cervical cancer screening behaviors still holds true for HPV testing in more recent years. This finding further signifies the need to increase the rates of HPV testing among females with diabetes in the U.S., especially because of the likelihood of a poorer cervical cancer prognosis with concurrent diabetes [11].

When comparing regional HPV testing rates to national rates, the South exhibited the lowest screening rates in all years. Various states in the Deep South demonstrated significantly lower screening rates for females with diabetes versus females without diabetes, such as Alabama, Georgia, Louisiana, South Carolina, and Tennessee. These differences in screening rates by diabetes diagnosis changed across years in most states in the Deep South. Notably, the marked difference persisted across all years in Louisiana. Overall, the states in the Deep South make up a majority of the “diabetes belt” with the highest prevalence of diabetes in the U.S. [3]. Therefore, the Deep South needs special attention, as the prevalence of diabetes, and by association, cancer, only appears to keep growing. Further, the higher cervical cancer incidence and mortality demonstrated in the Southern region of the U.S. speak to the need for additional attention in targeted cancer screening and other preventive measures [17,44].

Additionally, several points arise when discussing the impact of other factors on females performing appropriate HPV tests. We found Asian females to have lower odds of testing than Whites, and a previous study also reported lower cervical cancer screening rates among Asian Americans [45]. We found a higher likelihood of screening by non-Hispanic Black females than White females. In prior research, increased cervical cancer screening rates among Black females was shown to reduce the racial disparity in cervical cancer incidence [46], despite Black females still having a higher cervical cancer incidence compared to White females (8.3 vs. 7.4 per 100,000) [15]. In addition, changes in the cervical cancer incidence over time were likely affected by other influential factors, such as the HPV vaccine [46], access to care [47,48], health insurance coverage [47,48], geographic area [48], and sexual behavior [49]. For environmental factors, rural area residence participants could benefit from special attention in HPV test recommendations based on the low odds of testing found among females living in non-MSA areas in this study, especially considering that females in rural areas have been found to experience higher incidence rates of cervical cancer than those in urban areas [50]. Lastly, we also identified age as a factor impacting HPV testing, where females’ likeliness to perform HPV tests went down with increasing age after the age of 40. It is important to increase the uptake of HPV screening among females aged 40 and above given that cervical cancer diagnoses typically occur around the age of 50 [16].

Despite the concerns mentioned, it is also important to note that overall HPV testing rates have increased over time. This finding is similar to previous evaluations of co-testing, where rates of HPV test in this method increased after a change in ACS screening guidelines for cervical cancer in 2012 [40]. From 2012–2019, ACS guidelines recommended that persons aged 21–29 years should receive a Pap test every three years, and persons aged 30–65 should receive a Pap/HPV co-test every three years [51]. However, recent changes in guidelines for 2020 state that persons aged 25–65 should receive an HPV test alone every five years, so this preferred screening method may increase HPV screening from the most recent 2020 values reported in the present study. With the recent recommendation of HPV testing alone as a form of primary cervical cancer screening, healthcare providers must make efforts to adhere to new ACS guidelines. In particular, with the increased risk of cervical cancer for females with diabetes, special attention must be given to preventive cervical cancer screening practices among this population [10].

### Limitations

While we closely adhered to ACS guidelines, we could not make hard cutoffs for their age recommendations. All females within the 65–69 age category were included to capture females 65 years old. This led to the over-inclusion of females outside of the recommended ages in the ACS screening guidelines. With the self-reported responses in BRFSS, inaccurate classifications of variables, including diabetes diagnosis, could be present. Despite this concern, surveys with self-reported healthcare data, such as BRFSS, have still been found to have high reliability, particularly in preventive testing and diagnoses of chronic diseases/conditions [52]. It might also be important to consider differences in HPV testing rates by type of diabetes, but the BRFSS data do not currently differentiate between type 1 and type 2 diabetes. Additionally, cervical cancer screening practices may have been influenced by the 2012 ACS guidelines, where the preferred screening for cervical cancer was a Pap/HPV co-test for females ages 30–65 [51]. While HPV testing rates might be expected to closely align with Pap testing, this was not the case based on our results, where HPV testing rates are well below the Healthy People 2030 target cervical cancer screening rate of 84.3% in all years [41]. Lastly, caution must be used when interpreting the marked sub-group prevalence estimates by region or state among females with diabetes because of small sample size (unweighted frequency < 50) or large variability (95% confidence interval width >10) [38].

## 5. Conclusions

Females with diabetes are screening for cervical cancer with the HPV test less frequently than females without diabetes. Further, females living in the South reported the lowest rates of HPV testing, particularly in states in the Deep South. These populations of females with diabetes remain vulnerable to cervical cancer, so preventive measures must be taken through proper cervical cancer screenings. Future projections of diabetes prevalence, age, and regional disparities create a need for healthcare providers to adhere to the recent ACS guidelines that favor the primary HPV test. With the new recommendations, there may be a continued increase in HPV screening among persons with and without diabetes. However, critical work must be done to reach the currently projected goal for cervical cancer screenings of 84.3% from Healthy People 2030 [41], and special attention should be given to females with diabetes in the Deep South who are at greater risk. The practical implications from this study are as follows: (1) the overall utilization of the HPV test to screen for cervical cancer must be increased in the U.S., and (2) the population with the greatest need for increased HPV testing includes females with diabetes living in the Deep South.

## Figures and Tables

**Table 1:** Characteristics of Female Study Population, Stratified by Diabetes Diagnosis ^a^.

Characteristic	Females with Diabetes *n* = 41,442 Weighted *n* = 7,608,983	Females without Diabetes *n* = 320,104 Weighted *n* = 63,491,762	*p*-Value
	Weighted % (95% CI)	Weighted % (95% CI)	
Race			<0.001
White only, non-Hispanic	49.66 (48.55–50.78)	60.75 (60.38–61.12)	
Black only, non-Hispanic	15.41 (14.65–16.17)	11.64 (11.40–11.88)	
American Indian/Alaskan Native only	1.43 (1.23–1.63)	0.89 (0.83–0.94)	
Asian only, non-Hispanic	5.19 (4.41–5.97)	5.54 (5.30–5.79)	
Native Hawaiian, other Pacific Islander only, non-Hispanic	0.30 (0.21–0.38)	0.20 (0.17–0.23)	
Other race only, non-Hispanic	0.52 (0.36–0.67)	0.40 (0.36–0.43)	
Multiracial, non-Hispanic	1.47 (1.25–1.69)	1.31 (1.24–1.38)	
Hispanic	24.45 (23.28–25.61)	17.88 (17.54–18.21)	
Unknown	1.57 (1.25–1.89)	1.40 (1.31–1.49)	
Region			<0.001
South	38.90 (37.81–39.98)	35.74 (35.48–36.00)	
Northeast	16.64 (15.95–17.34)	18.44 (18.26–18.61)	
Midwest	18.96 (18.28–19.63)	20.64 (20.47–20.81)	
West	24.20 (23.11–25.30)	23.97 (23.72–24.22)	
U.S. territories	1.30 (1.18–1.41)	1.21 (1.18–1.24)	
Age			<0.001
25–29	4.51 (4.05–4.97)	13.67 (13.40–13.94)	
30–34	9.00 (8.31–9.68)	15.59 (15.30–15.88)	
35–39	9.40 (8.68–10.12)	12.69 (12.44–12.95)	
40–44	10.66 (9.93–11.39)	12.08 (11.83–12.33)	
45–49	9.94 (9.31–10.58)	9.75 (9.53–9.97)	
50–54	13.02 (12.18–13.85)	10.79 (10.56–11.01)	
55–59	14.58 (13.75–15.40)	9.43 (9.23–9.64)	
60–64	16.06 (15.28–16.84)	9.30 (9.11–9.50)	
65–69	12.83 (12.23–13.43)	6.69 (6.55–6.84)	
Education			<0.001
Never attended school or only kindergarten	0.58 (0.40–0.75)	0.28 (0.21–0.34)	
Elementary	8.53 (7.77–9.29)	3.69 (3.51–3.87)	
Some high school	11.87 (10.96–12.78)	6.77 (6.53–7.01)	
High school graduate	26.21 (25.27–27.14)	21.69 (21.39–22.00)	
Some college or technical school	31.05 (30.01–32.10)	30.48 (30.13–30.83)	
College graduate	21.51 (20.70–22.33)	36.91 (36.57–37.24)	
Unknown	0.25 (0.14–0.37)	0.18 (0.14–0.23)	
Metropolitan status			<0.001
In the center city of an MSA	11.56 (10.82–12.31)	9.03 (8.86–9.20)	
Outside the center city of an MSA but inside the county	7.06 (6.53–7.60)	6.13 (5.98–6.29)	
Inside a suburban county of the MSA	4.96 (4.57–5.35)	4.25 (4.14–4.36)	
Not in an MSA	5.75 (5.37–6.13)	4.26 (4.15–4.36)	
Unknown	70.67 (69.71–71.62)	76.33 (76.11–76.54)	
Employment			<0.001
Employed for wages	38.60 (37.53–39.68)	55.43 (55.06–55.80)	
Self-employed	5.71 (5.08–6.34)	8.45 (8.24–8.66)	
Out of work for ≥1 year	3.66 (3.24–4.09)	2.86 (2.72–3.00)	
Out of work for <1 year	3.89 (3.36–4.42)	3.73 (3.58–3.88)	
A homemaker	14.58 (13.62–15.53)	13.13 (12.85–13.41)	
A student	1.19 (0.89–1.49)	2.15 (2.03–2.26)	
Retired	13.18 (12.52–13.83)	7.49 (7.33–7.66)	
Unable to work	18.65 (17.86–19.43)	5.98 (5.81–6.15)	
Unknown	0.54 (0.40–0.69)	0.78 (0.69–0.86)	
Income			<0.001
<$10,000	8.61 (7.97–9.24)	4.64 (4.48–4.81)	
$10,000–$14,999	7.47 (6.88–8.06)	3.72 (3.58–3.87)	
$15,000–$19,999	9.74 (9.03–10.45)	5.70 (5.52–5.87)	
$20,000–$24,999	9.68 (9.01–10.35)	7.01 (6.82–7.21)	
$25,000–$34,999	8.98 (8.35–9.60)	7.69 (7.50–7.89)	
$35,000–$49,999	9.87 (9.25–10.48)	10.53 (10.30–10.76)	
$50,000–$74,999	11.13 (10.46–11.81)	13.27 (13.02–13.51)	
≥$75,000	19.34 (18.46–20.21)	34.11 (33.77–34.45)	
Unknown	15.19 (14.31–16.06)	13.33 (13.07–13.59)	
Health insurance			0.178
Yes	87.13 (86.26–88.00)	87.58 (87.30–87.85)	
No	12.47 (11.61–13.32)	12.16 (11.88–12.44)	
Unknown	0.41 (0.20–0.61)	0.26 (0.22–0.30)	
Marital status			<0.001
Married	51.42 (50.29–52.54)	56.97 (56.60–57.33)	
Divorced	15.32 (14.52–16.12)	11.61 (11.39–11.83)	
Widowed	7.78 (7.19–8.38)	3.41 (3.30–3.52)	
Separated	4.90 (4.42–5.38)	3.20 (3.06–3.33)	
Never married	15.59 (14.83–16.35)	18.66 (18.37–18.95)	
Member of unmarried couple	4.51 (4.01–5.02)	5.68 (5.49–5.86)	
Unknown	0.48 (0.24–0.72)	0.48 (0.42–0.54)	
Veteran status			0.005
Yes	2.03 (1.71–2.35)	2.47 (2.36–2.59)	
No	97.93 (97.61–98.25)	97.45 (97.33–97.57)	
Unknown	0.04 (0.02–0.06)	0.08 (0.06–0.10)	
General health status			<0.001
Excellent	6.48 (5.91–7.04)	22.47 (22.17–22.78)	
Very good	17.45 (16.66–18.24)	34.82 (34.48–35.16)	
Good	35.78 (34.70–36.86)	29.75 (29.40–30.10)	
Fair	27.91 (26.91–28.91)	10.18 (9.94–10.41)	
Poor	12.03 (11.19–12.86)	2.60 (2.49–2.72)	
Unknown	0.36 (0.23–0.49)	0.17 (0.13–0.21)	
Difficulty visiting doctor’s office alone			<0.001
Yes	14.83 (14.08–15.58)	5.57 (5.40–5.73)	
No	84.78 (84.02–85.54)	94.26 (94.09–94.42)	
Unknown	0.39 (0.22–0.56)	0.18 (0.15–0.21)	
Year			0.010
2016	37.94 (36.89–38.98)	39.35 (39.03–39.68)	
2018	33.59 (32.53–34.64)	31.93 (31.61–32.26)	
2020	28.48 (27.39–29.56)	28.72 (28.38–29.05)	

^a^ Abbreviations: BRFSS = Behavioral Risk Factor Surveillance System; MSA = metropolitan statistical area; VA = Veterans Affairs. Unknown indicates responses of don’t know/Not sure/Refused or not asked/missing.

**Table 2:** Factors Associated with HPV Testing Behaviors among Female Study Population ^a^.

Characteristic	Prevalence Screening with HPV Test (%) (95% Confidence Interval) *n* = 361,546 Weighted *n* = 71,100,745	Adjusted Odds Ratio (95% Confidence Interval) *n* = 361,546 Weighted *n* = 71,100,745
Diabetes		
Yes	37.95 (36.87–39.04)	0.934 (0.886–0.985) **
No	46.21 (45.84–46.58)	Ref
Race		
White only, non-Hispanic	44.35 (43.98–44.73)	Ref
Black only, non-Hispanic	52.60 (51.58–53.61)	1.352 (1.287–1.420) **
American Indian or Alaskan Native only	47.11 (44.15–50.06)	1.150 (1.007–1.314) **
Asian only, non-Hispanic	34.70 (32.57–36.83)	0.570 (0.514–0.631) **
Native Hawaiian, other Pacific Islander only, non-Hispanic	44.41 (38.62–50.20) ^b^	0.920 (0.730–1.159)
Other race only, non-Hispanic	48.91 (44.53–53.28)	1.177 (0.980–1.414)
Multiracial, non-Hispanic	57.52 (55.11–59.93)	1.408 (1.268–1.563) **
Hispanic	46.09 (45.03–47.15)	1.221 (1.155–1.291) **
Unknown	42.31 (39.16–45.46)	1.003 (0.872–1.155)
Region		
South	44.69 (44.06–45.32)	Ref
Northeast	46.14 (45.45–46.84)	1.138 (1.093–1.184) **
Midwest	44.67 (44.08–45.27)	1.015 (0.978–1.053)
West	46.17 (45.34–47.01)	1.115 (1.066–1.167) **
U.S. territories	46.34 (44.67–48.01)	1.033 (0.942–1.133)
Age		
25–29	56.22 (55.20–57.25)	Ref
30–34	58.53 (57.53–59.52)	1.114 (1.049–1.183) **
35–39	54.87 (53.82–55.91)	0.972 (0.914–1.035)
40–44	50.77 (49.70–51.84)	0.810 (0.759–0.864) **
45–49	46.20 (45.10–47.30)	0.667 (0.624–0.713) **
50–54	39.46 (38.44–40.48)	0.501 (0.469–0.536) **
55–59	33.76 (32.76–34.77)	0.386 (0.360–0.414) **
60–64	28.24 (27.34–29.15)	0.297 (0.276–0.320) **
65–69	21.61 (20.72–22.49)	0.207 (0.190–0.226) **
Education		
Never attended school or only kindergarten	24.16 (17.63–30.69) ^b^	0.419 (0.282–0.623) **
Elementary	30.51 (28.52–32.49)	0.561 (0.501–0.629) **
Some high school	39.58 (37.89–41.27)	0.720 (0.664–0.782) **
High school graduate	40.87 (40.14–41.60)	0.808 (0.774–0.843) **
Some college or technical school	47.61 (46.95–48.26)	0.967 (0.932–1.003)
College graduate	49.37 (48.87–49.87)	Ref
Unknown	33.48 (24.57–42.39) ^b^	0.669 (0.377–1.185)
Metropolitan status		
In the center city of an MSA	37.88 (36.84–38.92)	1.247 (1.159–1.342) **
Outside the center city of an MSA but inside the county	37.73 (36.40–39.07)	1.232 (1.132–1.340) **
Inside a suburban county of the MSA	38.51 (37.13–39.89)	1.225 (1.124–1.335) **
Not in an MSA	31.91 (30.69–33.12)	Ref
Unknown	48.04 (47.62–48.46)	1.318 (1.239–1.402) **
Employment		
Employed for wages	49.62 (49.16–50.08)	Ref
Self-employed	44.89 (43.61–46.18)	0.993 (0.937–1.053)
Out of work for ≥1 year	43.96 (41.66–46.27)	0.997 (0.902–1.103)
Out of work for <1 year	50.82 (48.87–52.77)	1.067 (0.980–1.163)
A homemaker	41.17 (40.08–42.25)	0.880 (0.835–0.928) **
A student	53.61 (50.94–56.29)	0.912 (0.811–1.025)
Retired	26.41 (25.43–27.39)	0.968 (0.903–1.039)
Unable to work	38.86 (37.65–40.08)	0.864 (0.804–0.929) **
Unknown	39.76 (34.40–45.11) ^b^	0.822 (0.664–1.017)
Income		
<$10,000	40.68 (39.10–42.27)	0.683 (0.625–0.746) **
$10,000–$14,999	41.34 (39.57–43.10)	0.759 (0.691–0.833) **
$15,000–$19,999	44.15 (42.71–45.60)	0.815 (0.755–0.879) **
$20,000–$24,999	46.36 (45.01–47.71)	0.871 (0.814–0.932) **
$25,000–$34,999	45.68 (44.44–46.93)	0.844 (0.791–0.899) **
$35,000–$49,999	47.20 (46.10–48.31)	0.894 (0.844–0.946) **
$50,000–$74,999	46.05 (45.09–47.00)	0.852 (0.811–0.894) **
≥$75,000	49.51 (48.92–50.10)	Ref
Unknown	35.86 (34.88–36.84)	0.663 (0.627–0.702) **
Health insurance		
Yes	46.05 (45.68–46.41)	Ref
No	40.50 (39.35–41.65)	0.774 (0.733–0.818) **
Unknown	30.99 (24.87–37.12) ^b^	0.600 (0.444–0.811) **
Marital status		
Married	42.62 (42.15–43.09)	Ref
Divorced	47.06 (46.12–48.00)	1.413 (1.347–1.481) **
Widowed	29.55 (28.12–30.97)	1.065 (0.984–1.153)
Separated	48.62 (46.66–50.57)	1.356 (1.243–1.479) **
Never married	52.63 (51.80–53.46)	1.159 (1.107–1.213) **
Member of unmarried couple	54.32 (52.71–55.92)	1.338 (1.245–1.438) **
Unknown	41.43 (35.25–47.62) ^b^	1.125 (0.857–1.476)
Veteran status		
Yes	57.12 (54.89–59.34)	1.387 (1.254–1.534) **
No	45.05 (44.69–45.40)	Ref
Unknown	29.37 (16.25–42.50) ^b^	0.699 (0.364–1.343)
General health status		
Excellent	48.57 (47.80–49.33)	1.027 (0.986–1.069)
Very good	47.74 (47.17–48.30)	Ref
Good	43.03 (42.36–43.70)	0.858 (0.826–0.892) **
Fair	41.45 (40.42–42.49)	0.870 (0.820–0.924) **
Poor	37.82 (35.89–39.75)	0.795 (0.718–0.880) **
Unknown	31.32 (23.38–39.27) ^b^	0.599 (0.400–0.896) **
Difficulty visiting doctor’s office alone		
Yes	43.83 (42.49–45.17)	1.040 (0.968–1.117)
No	45.45 (45.09–45.81)	Ref
Unknown	37.88 (30.82–44.94) ^b^	0.946 (0.680–1.318)
History of myocardial infarction		
Yes	38.50 (36.05–40.95)	1.064 (0.942–1.200)
No	45.49 (45.13–45.84)	Ref
Unknown	36.17 (29.49–42.85) ^b^	0.973 (0.719–1.317)
History of coronary artery disease		
Yes	37.62 (35.15–40.09)	1.059 (0.936–1.199)
No	45.52 (45.17–45.88)	Ref
Unknown	33.06 (26.21–39.92) ^b^	0.887 (0.644–1.223)
History of stroke		
Yes	41.07 (38.62–43.52)	1.111 (0.992–1.244)
No	45.43 (45.08–45.79)	Ref
Unknown	36.31 (28.10–44.53) ^b^	0.829 (0.571–1.203)
History of asthma		
Yes	50.43 (49.55–51.30)	1.170 (1.122–1.220) **
No	44.39 (44.00–44.77)	Ref
Unknown	41.44 (33.67–49.21) ^b^	0.996 (0.704–1.408)
History of skin cancer		
Yes	38.97 (37.53–40.42)	1.075 (1.006–1.149) **
No	45.59 (45.23–45.95)	Ref
Unknown	36.45 (27.60–45.29) ^b^	0.837 (0.557–1.257)
History of non-skin cancer		
Yes	47.42 (45.92–48.92)	1.523 (1.427–1.625) **
No	45.20 (44.84–45.56)	Ref
Unknown	52.80 (45.56–60.03) ^b^	1.717 (1.307–2.254) **
History of COPD		
Yes	41.61 (40.21–43.00)	1.082 (1.010–1.160) **
No	45.58 (45.22–45.95)	Ref
Unknown	34.50 (27.83–41.17) ^b^	0.820 (0.615–1.093)
History of arthritis		
Yes	40.19 (39.52–40.86)	1.117 (1.073–1.162) **
No	46.87 (46.46–47.28)	Ref
Unknown	34.24 (29.70–38.78)	0.680 (0.549–0.844) **
History of depression		
Yes	50.15 (49.46–50.84)	1.232 (1.188–1.278) **
No	43.94 (43.53–44.34)	Ref
Unknown	42.13 (35.67–48.59) ^b^	0.976 (0.751–1.268)
History of chronic kidney disease		
Yes	42.14 (39.86–44.41)	1.100 (0.992–1.219)
No	45.41 (45.05–45.76)	Ref
Unknown	42.50 (30.59–54.40) ^b^	1.205 (0.707–2.055)
Year		
2016	40.63 (40.10–41.16)	Ref
2018	48.00 (47.39–48.61)	1.363 (1.316–1.411) **
2020	48.76 (48.05–49.47)	1.378 (1.326–1.432) **

** *p*-value < 0.05. ^a^ Abbreviations: BRFSS = Behavioral Risk Factor Surveillance System; MSA = metropolitan statistical area; VA = Veterans Affairs; COPD = chronic obstructive pulmonary disease. Unknown indicates responses of don’t know/Not sure/Refused or not asked/missing. Reference group chosen as the modal category for all characteristics except age (youngest age selected as ref.) and metropolitan status (category representing rural selected as ref.). ^b^ Use caution when interpreting prevalence estimates within this sub-group; 95% confidence interval width >10.

## Data Availability

Publicly available datasets were analyzed in this study. This data can be found here: https://www.cdc.gov/brfss/annual_data/annual_data.htm, accessed on 14 December 2021.
